# The diagnostic accuracy of three rapid diagnostic tests for typhoid fever at Chittagong Medical College Hospital, Chittagong, Bangladesh

**DOI:** 10.1111/tmi.12559

**Published:** 2015-07-15

**Authors:** Rapeephan R. Maude, Hanna K. de Jong, Lalith Wijedoru, Masako Fukushima, Aniruddha Ghose, Rasheda Samad, Mohammed Amir Hossain, Mohammed Rezaul Karim, Mohammed Abul Faiz, Christopher M. Parry, CMCH Typhoid Study Group, Abdullah Abu Sayeed, Uddin Hasan, Wirichada Pan‐Ngum, Thomas W. van der Vaart, Asok Kumar Dutta, Nasir Uddin Mahmud, Murad Hero, Nafiz Iqbal, Zabeen Chaudhury, Tran Vu Thieu Nga, Pham Thanh Duy, Voong Vinh Phat, Richard J. Maude, Stephen Baker, W. Joost Wiersinga, Tom van der Poll, Nicholas P. Day, Arjen M. Dondorp

**Affiliations:** ^1^ Mahidol‐Oxford Tropical Medicine Research Unit (MORU)Faculty of Tropical MedicineMahidol UniversityBangkokThailand; ^2^ Department of Internal MedicineDivision of Infectious Diseases and Center for Infection and Immunity Amsterdam (CINIMA)AmsterdamThe Netherlands; ^3^ Center for Experimental Molecular Medicine (CEMM)Academic Medical CenterUniversity of AmsterdamAmsterdamThe Netherlands; ^4^ Clinical SciencesLiverpool School of Tropical MedicineLiverpoolUK; ^5^ Chittagong Medical College HospitalChittagongBangladesh; ^6^ Centre for Specialized Care and ResearchChittagongBangladesh; ^7^ London School of Hygiene and Tropical MedicineLondonUK; ^8^ School of Tropical Medicine and Global HealthNagasaki UniversityNagasakiJapan

**Keywords:** typhoid fever, *Salmonella enterica* serovar Typhi, blood culture, real‐time PCR, rapid diagnostic tests, diagnostic accuracy, fièvre typhoïde, *Salmonella enterica* sérotype typhi, culture de sang, PCR en temps réel, tests de diagnostic rapide, précision diagnostique

## Abstract

**Objective:**

To determine the diagnostic accuracy of three rapid diagnostic tests (RDTs) for typhoid fever in febrile hospitalised patients in Bangladesh.

**Methods:**

Febrile adults and children admitted to Chittagong Medical College Hospital, Bangladesh, were investigated with Bact/Alert^®^ blood cultures and real‐time PCR to detect *Salmonella enterica* Typhi and Paratyphi A and assays for *Rickettsia*, leptospirosis and dengue fever. Acute serum samples were examined with the LifeAssay (LA) Test‐it™ Typhoid IgM lateral flow assay detecting IgM antibodies against *S. *Typhi O antigen, CTKBiotech Onsite Typhoid IgG/IgM Combo Rapid‐test cassette lateral flow assay detecting IgG and IgM antibodies against *S. *Typhi O and H antigens and SD Bioline line assay for IgG and IgM antibodies against *S. *Typhi proteins.

**Results:**

In 300 malaria smear‐negative febrile patients [median (IQR) age of 13.5 (5–31) years], 34 (11.3%) had confirmed typhoid fever: 19 positive by blood culture for *S. *Typhi (three blood PCR positive) and 15 blood culture negative but PCR positive for *S. *Typhi in blood. The respective sensitivity and specificity of the three RDTs in patients using a composite reference standard of blood culture and/or PCR‐confirmed typhoid fever were 59% and 61% for LifeAssay, 59% and 74% for the CTK IgM and/or IgG, and 24% and 96% for the SD Bioline RDT IgM and/or IgG. The LifeAssay RDT had a sensitivity of 63% and a specificity of 91% when modified with a positive cut‐off of ≥2+ and analysed using a Bayesian latent class model.

**Conclusions:**

These typhoid RDTs demonstrated moderate diagnostic accuracies, and better tests are needed.

## Abstract (fr)

**Objectif:**

Déterminer la précision diagnostique de trois tests de diagnostic rapide (TDR) pour la fièvre typhoïde chez les patients fébriles hospitalisés au Bangladesh.

**Méthodes:**

Les adultes et enfants fébriles admis à l'hôpital de Chittagong Medical College, au Bangladesh ont été investigués avec: BactAlert^®^ pour les cultures de sang, la PCR en temps réel pour détecter *S. enterica typhi* et *paratyphi A* et les tests pour *Rickettsia*, la leptospirose et la fièvre de la dengue. Des échantillons de sérum prélevé en phase aiguë ont été examinés avec le test LifeAssay (LA) Test‐it^™^ Typhoïd IgM à écoulement latéral détectant les anticorps IgM contre l'antigène O de *S. typhi*, le test CTKBiotech Onsite Typhoïd IgG/IgM Combo Rapid‐test cassette à écoulement latéral détectant les anticorps IgG et IgM contre antigènes O et H de *S. typhi* et le test SD Bioline pour les anticorps IgG et IgM contre les protéines de *S. typhi*.

**Résultats:**

Sur 300 patients fébriles avec à frottis négatif pour le paludisme (âge médian (IQR): 13,5 ans (5 à 31)), 34 (11,3%) ont eu la fièvre typhoïde confirmée: 19 positifs par la culture de sang pour *S. typhi* (3 échantillons de sang positif avec la PCR) et 15 hémocultures négatives, mais positives pour *S. typhi* par la PCR sur le sang. La sensibilité et la spécificité respectives des trois TDR chez les patients, en utilisant un standard de référence composé de la culture de sang et/ou la PCR pour confirmer la fièvre typhoïde, était de 36% et 89% pour le LifeAssay, 54% et 74% pour le CTK IgM et/ou IgG et 21% et 97% pour le SD Bioline IgM et/ou IgG. Le TDR LifeAssay avait une sensibilité de 63% et une spécificité de 91% lorsqu'il était modifié pour un seuil de positivité fixé à ≥ 2+ et analysé en utilisant un modèle de structure latente bayésienne.

**Conclusions:**

Ces TDR pour la typhoïde ont démontré des précisions diagnostiques modérées; de meilleurs tests sont donc toujours nécessaires.

## Abstract (es)

**Objetivo:**

Determinar la precisión diagnóstica de tres pruebas de diagnóstico rápido (PDR) para la fiebre tifoidea en pacientes hospitalizados y con fiebre en Bangladesh.

**Métodos:**

A pacientes adultos y niños, febriles y hospitalizados en el Hospital Universitario de Chittagong, se les realizaron las siguientes pruebas: hemocultivo con BactAlert^®^; PCR a tiempo real para detectar *S.enterica typhi* y *paratyphi* A; y pruebas para *Rickettsia*, leptospirosis y fiebre del dengue. A las muestras de suero de pacientes en fase aguda se les realizaron las siguientes pruebas: detección de anticuerpos IgM frente a al antígeno O de *S.typhi* con el inmunoensayo de flujo lateral LifeAssay (LA) Test‐it^™^; la prueba rápida CTKBiotech Onsite Tifoidea IgG/IgM Combo en casete para la detección de anticuerpos IgG e IgM frente a los antígenos O y H de *S. typhi*; y la prueba rápida SD Bioline para detección de anticuerpos IgG e IgM frente a proteínas de *S. typhi*.

**Resultados:**

De 300 pacientes febriles con resultados negativos para malaria (edad media de 13.5 (5‐31) años), 34 (11.3%) tenían fiebre tifoidea confirmada: 19 con cultivo positivo para *S. typhi* (3 con PCR positiva en sangre); 15 con hemocultivo negativo pero con resultados positivo para la PCR de *S. typhi* en sangre. La sensibilidad y especificidad respectiva para las tres PDRs en pacientes, utilizando un estándar de referencia compuesto de fiebre tifoidea confirmada por hemocultivo y/o PCR eran 36% y 89% para LifeAssay, 54% y 74% para CTK IgM y/o IgG y 21% y 97% para la PDR de SD Bioline IgM y/o IgG. La PDR de LifeAssay tenía una sensibilidad del 63% y una especificidad del 91% cuando se modificaba con un punto de corte positivo de ≥2+ y se analizaba utilizando un modelo Bayesiano de clases latentes.

**Conclusiones:**

Estas PDR para fiebre tifoidea demuestran una precisión diagnóstica moderada y se necesitan mejores pruebas.

## Background

Typhoid (enteric) fever is a common infection in adults and children in Bangladesh 1, 2, 3. Antimicrobial resistance to standard treatments for *Salmonella enterica* serovars Typhi and Paratyphi A infections is widespread 4, 5. In routine practice, a diagnosis of typhoid fever is rarely confirmed as diagnostic tests are unavailable or have limited diagnostic accuracy. Blood culture is the commonest reference standard test but has an estimated sensitivity of between 40% and 80% 6. Blood culture sensitivity is limited by low number of bacteria circulating in the blood, small volumes of blood taken for culture and prior antimicrobial therapy 7. Bone marrow culture is more sensitive than blood culture, because of the higher number of bacteria present, and can remain positive several days after effective antimicrobial treatment has started, yet is rarely used except in a research context 8, 9. Nucleic acid amplification methods are available but demonstrate variable sensitivities and specificities 6, 10. The Widal test is still used but as it is based on cross‐reactive antigens, it lacks sensitivity and specificity 11, 12.

Clinicians often use rapid diagnostic tests (RDTs) to diagnose typhoid. A number of typhoid fever RDTs are commercially available but have variable performance 6, 13. We have measured the diagnostic accuracy of three commercially available typhoid RDTs as part of a fever study conducted at Chittagong Medical College Hospital (CMCH) in Bangladesh. We used Bayesian latent class models (LCM) to evaluate the results in the absence of an adequate reference standard 14, 15, 16.

## Patients and methods

### Ethics statement

The study was conducted according to the principles expressed in the Declaration of Helsinki. The Bangladesh National Research Ethical Committee (BMRC/NREC/2010‐2013/1543), the Chittagong Medical College Hospital Ethical Committee, the Oxford Tropical Research Ethics Committee (Oxtrec 53‐09) and the Research Ethics committee of the Liverpool School of Tropical Medicine gave ethical approval for the study. Informed written or thumbprint consent was taken from the subject, their parent or caretaker for all cases and controls.

### Study site and patients

Chittagong Medical College Hospital is a 1000‐bed teaching hospital serving the population of Chittagong and surrounding province. Adults and children (age > 6 months) consecutively admitted to the adult and paediatric wards at CMCH with a documented axillary temperature of ≥38 °C in the period up to 48 h after admission and history of fever of <2 weeks were eligible for entry to the study. We also recruited 38 healthy adult controls from among the hospital staff to determine background levels of antibodies, as determined by the RDTs, in this population. Study recruitment was between January and June 2012.

### Clinical procedures

Demographic and clinical information was recorded on a study case report form at the time of study admission. Blood was collected at the time of study admission for complete blood count, renal function, aspartate transaminase (AST), alanine transaminase (ALT), blood culture, EDTA whole blood for PCR, serum for the RDTs and a malaria smear. The samples for PCR and the RDTs were frozen at −20 °C and tested later at Oxford Clinical Research Unit (OUCRU), Ho Chi Minh City, Vietnam. A diagnosis or differential diagnosis was proposed and recorded by the research team at the time of discharge or death including a judgment, based on the blood culture results and assessment by the research team, as to whether the clinical features, laboratory results and inpatient progress of the patient were consistent with typhoid fever. These features included the following: a febrile illness of >3 days duration, the presence of abdominal symptoms (abdominal pain, diarrhoea or constipation), a documented fever of ≥39 °C; hepatomegaly and/or splenomegaly, a low or normal white cell count, elevation of liver enzymes (AST, ALT) 2–3 times above the normal range, a slow defervescence with ceftriaxone treatment (the standard antimicrobial used for hospital admitted febrile patients); no alternative confirmed diagnosis established 17.

Demographic and clinical information from the healthy control subjects was also recorded on the study case report form and blood collected for complete blood count, EDTA whole blood for PCR, and serum for the RDTs.

### Laboratory investigations and microbiology

A volume of blood, 5–12 ml in adults and 1–12 ml in children, was inoculated into a Bact/Alert^®^ FA and PF blood culture bottles and incubated in the Bact/Alert^®^ automated system (bioMérieux, Marcy l'Etoile, France) for 5 days. A Gram‐stained smear was prepared from the broth of blood bottles that were culture positive and was subcultured onto 5% sheep blood agar and chocolate agar incubated in a candle jar and MacConkey agar incubated in air for 48 h (all media Oxoid, Basingstoke, UK). Bacterial isolates were identified by standard methods including biochemical test using API test strips (bioMérieux) and agglutination with specific antisera (Bio‐Rad, Hertfordshire, UK). All media and tests were subject to regular internal quality assessment. Bacterial isolates were stored on beads in glycerol at −80 °C and later transferred to the OUCRU, Vietnam, for reconfirmation of their identification.

DNA extraction was performed at OUCRU, Vietnam, from the stored whole blood in EDTA using a QIAmp^®^ DNA mini kit (QIAGEN, UK). The volume of blood used to extract DNA was usually 2 ml from adults and 1 ml from children. The real‐time PCR method previously published by Nga *et al*. was followed using 25 μl reactions containing 5 μl of extracted DNA targeting *STY0201* (Putative fimbrial adhesion in *S*. Typhi CT18) or *SSPA2308* (hypothetical protein in SPA AKU‐12601) for the detection of *S*. Typhi and *S*. Paratyphi A in blood 10. Probe‐based real‐time PCR was also performed on DNA extracted from blood to detect *Leptospira* spp. 18, *Rickettsia typhi* 19 and *Orientia tsutsugamushi* 20. Low‐positive plasmid controls determined adequate detection limits of each assay. Serum was analysed by ELISA for dengue NS1 antigen using a PanBio Kit (PanBio, Australia) according to the manufacturer's instructions.

The Life Assay Test‐it™ Typhoid IgM (Life Assay Diagnostics (Pty), Cape Town, South Africa) is a lateral flow assay detecting IgM antibodies against *S. enterica* Typhi (ST) O antigen and takes 15 min to perform and read. The LifeAssay result for each sample was graded as recommended using the band intensity from 0 to 4+. The SD Bioline test (Standard Diagnostics Inc., Gyeonggi, Korea) uses an immunochromatographic method to visually and qualitatively detect IgM and IgG antibodies to serotype Typhi antigens (unspecified) that are indirectly labelled with colloidal gold (via an antibody). The antigen‐bound antibodies are captured by anti‐IgM or anti‐IgG antibodies. The test takes 30 min to perform and read. The CTKBiotech Onsite Typhoid IgG/IgM Combo Rapid‐test cassette (CTK Biotech, Inc., San Diego, CA, USA) is a lateral flow assay that detects IgG and IgM antibodies against recombinant O and H *S*. Typhi antigens. The test can be performed and read in 15 min. The SD Bioline test and CTKBiotech Onsite Typhoid IgG/IgM Combo kits are available and used in Chittagong diagnostic laboratories. The tests were performed on the stored serum at OUCRU, Vietnam, according to the manufacturer's instructions. The real‐time PCR and RDTs were conducted blind to the other laboratory results including blood culture results and clinical data.

### Analysis

The Standards for Reporting of Diagnostic Accuracy reporting guidelines were followed 21. The sensitivity, specificity and predictive values with 95% confidence intervals were calculated. Laboratory‐confirmed typhoid cases were defined if the patient's blood culture and/or blood PCR was positive for Typhi or Paratyphi A (composite reference standard positive). Cases that were not typhoid were defined if the patient's blood culture and blood PCR was negative for Typhi or Paratyphi A (composite reference standard negative).

Analysis was performed using SPSS version 22 (IBM SPSS Statistics, Chicago, IL, USA). The Bayesian LCM as described by Dendukuri and Joseph 22 was used to approximate the prevalence, sensitivities and specificities of all tests. The Bayesian LCM does not assume that any test is perfect, but considers that each test could be imperfect in diagnosing the true disease status. The true disease status of the patient population is then defined on the basis of overall prevalence (the probability that a patient with suspected typhoid fever is truly infected with *S*. Typhi). LCMs estimate prevalence and accuracy of each test based on the observed frequency of the possible combinations of test results. The model assumed that no other prior information (non‐informative priors) about the unknown parameters (prevalence, sensitivities and specificities) was available except the specificity of blood culture was set at 100% and that there was a correlation between the blood culture and PCR result. The median values of all the parameters were reported together with the 95% credible intervals 23. The Bayesian LCM was run using WinBUGS 1.4 (Medical Research Council, and Imperial College London, UK).

## Results

### Clinical features

**Figure 1:**
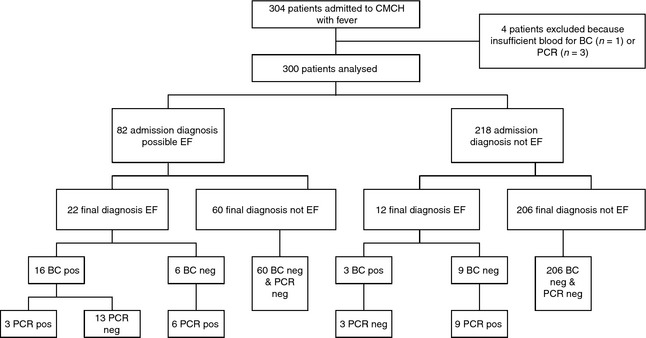
Flowchart of the 304 febrile patients admitted to the study and the analysis results.

The flow of patients in the study is illustrated in Figure 1. We enrolled 304 eligible febrile patients admitted to CMCH. In one patient, it was not possible to take blood, and in three patients, who were blood culture negative, there was insufficient blood for the PCR assay. These four patients were excluded from this analysis. Of the 300 patients analysed, 156 were children (age ≤ 15 years) and 144 were adults. The median (interquartile range, range) age was 13.5 (5.0–31.0, 0.5–89) years, and the median (IQR, range) duration of illness before admission was 5 (2–8, 1–14) days. A total of 29 (9.7%) patients died during their hospital admission including two with typhoid fever (one blood culture and one PCR confirmed).

Typhoid fever was confirmed in 34 patients (11.3%, 95% CI; 8.2–15.5). Nineteen (6.3%) had a positive blood culture for *S. *Typhi (three of which were also blood PCR positive) and 15 (5.0%) had a negative blood culture but tested PCR positive for *S. *Typhi in blood. Eighteen (6.0%) additional patients had a clinical syndrome suggestive of typhoid fever but tested negative by blood culture and PCR. No patient was positive for *S. *Paratyphi A by blood culture or by blood PCR. Diagnoses in the 248 other patients were a non‐specific febrile illness in 48 (16.0%), lower respiratory tract infection in 48 (16.0%), CNS infections in 37 (12.3%), urinary sepsis in 23 (7.7%), upper respiratory tract infection in 21 (7.0%), diarrhoea or dysentery in 21 (7.0%), malaria suspected in seven (2.3%) (none were microscopy proven probably because of prior treatment), liver conditions in seven (2.3%), skin or soft tissue infection in four (1.3%), dengue in 3 (1.0%), severe sepsis in two (0.7%), septic arthritis in two (0.7%), nephrotic syndrome in two (0.7%), murine typhus in two (0.7%), scrub typhus in one (0.3%), leptospirosis in one (0.3%), dental abscess in one (0.3%) and non‐infection conditions in 18 (6.0%). Pathogens detected by blood culture and/or by blood PCR were *Staphylococcus aureus* (2), *Streptococcus pneumoniae* (1), *Streptococcus acidominimus* (1), *Enterococcus gallinarum* (1), *Escherichia coli* (2), *Klebsiella pneumoniae* (1), *Enterobacter cloacae* (2), *Acinetobacter* spp. (1), *Burkholderia cepacia* (3), dengue (2), *R. typhi* (2) and *O. tsutsugamushi (1)*.

The patients were stratified into two groups for subsequent analysis: blood culture positive and/or PCR positive for *S. *Typhi (*n* = 34) and blood culture and PCR negative for *S. *Typhi (*n* = 266). The clinical features and laboratory results from these patient groups are in Table 1. Patients with typhoid were more commonly adults, with a longer duration of illness prior to admission, more likely to have recently taken an antimicrobial, more likely to have a headache and diarrhoea but less likely to have had a seizure. The white cell count and platelet count were lower in those with typhoid than those without typhoid and the liver enzymes. ALT and AST were more likely to be elevated. In adults, the median volumes of blood taken for culture were comparable between the patient groups, whereas larger blood volumes were taken for culture in those with typhoid compared with those without typhoid in the children. Among patients with confirmed typhoid, 7 of 19 (37%) with a positive blood culture had taken an antimicrobial active against local strains of *S. *Typhi before admission compared with 8 of 15 (53%) with a negative blood culture but positive by blood PCR (*P* = 0.489).

**Table 1:** Demographic, clinical and laboratory features of studied patients (proportions are expressed as number (%) and continuous variables as median (interquartile range)

Variable	All patients	Typhoid fever [BC positive and/or PCR positive]	Not typhoid fever [BC negative and PCR negative]	*P*‐value^a^
Number	300	34	266	
Age (years)	13.5 (5–31)	25 (20–32)	10 (4–30)	0.002
Sex (male)	173 (58)	20 (59)	153 (58)	1.00
Duration of illness (days)	5 (2–8)	7 (5–13)	4 (2–7)	<0.001
Recent antimicrobial	151 (50)	26 (77)	125 (47)	0.002
Recent antimicrobial active against *S*.Typhi^b^	73 (24)	15 (46)	58 (22)	0.005
Headache	106 (35)	21 (62)	85 (32)	0.001
Abdominal pain	96 (32)	15 (44)	81 (31)	0.108
Diarrhoea	58 (19)	11 (32)	47 (18)	0.041
Constipation	41 (14)	6 (18)	35 (13)	0.435
Vomiting	126 (42)	19 (56)	107 (40)	0.082
Cough	122 (41)	12 (35)	110 (41)	0.498
Shortness of breath	14 (5)	2 (6)	12 (5)	0.721
Seizures	53 (18)	1 (3)	52 (20)	0.015
Temperature ≥39 °C	56 (33)	8 (30)	48 (33)	0.707
Jaundice	22 (7)	4 (12)	18 (7)	0.292
Hepatomegaly	37 (12)	6 (18)	31 (12)	0.402
Splenomegaly	15 (5)	0 (0)	15 (16)	0.233
Altered consciousness	59 (20)	2 (6)	57 (21)	0.037
Haemoglobin (g/dl)	11.4 (10.2–12.8)	11.6 (10.4–12.7)	11.4 (10.2–12.8)	0.798
White cell count (× 10^9^/l)	12.0 (8.0–16.0)	7.1 (5.0–11.1)	12.0 (8.5–16.0)	<0.001
Neutrophils (× 10^9^/l)	8.5 (5.0–12.2)	4.9 (3.1–7.5)	8.9 (5.6–12.6)	<0.001
Lymphocytes (× 10^9^/l)	2.2 (1.4–3.2)	1.5 (1.0–2.4)	2.2 (1.5–3.3)	<0.001
Monocytes (× 10^9^/l)	0.3 (0.2–0.4)	0.2 (0.1–0.3)	0.3 (0.2–0.4)	<0.001
Eosinophils (× 10^9^/l)	0.14 (0.08–0.24)	0.08 (0.04–0.15)	0.16 (0.09–0.24)	<0.001
Platelet count (× 10^6^/l)	240 (180–338)	190 (158–233)	240 (180–346)	<0.001
AST (IU/l)	37 (25–68)	62 (33–125)	35 (23–60)	<0.001
ALT (IU/l)	30 (25–55)	55 (30–100)	30 (19–49)	<0.001
Volume of blood taken for culture (age < 5 years) (ml)	2.2 (1.7–2.9)	–	2.2 (1.7–2.9)	–
Volume of blood taken for culture (age 5–9 years) (ml)	5.2 (4.2–5.8)	8.7 (8.2–8.7)	5.1 (4.2–5.8)	0.049
Volume of blood taken for culture (age 10–15 years) (ml)	6.6 (5.5–9.9)	7.6 (5.9–9.9)	6.5 (5.4–10.2)	0.837
Volume of blood taken for culture (age ≥ 16 years) (ml)	9.2 (8.4–10.3)	9.3 (8.4–10.7)	9.2 (8.5–10.2)	0.580
Death in hospital	29 (9.7)	2 (5.9)	27 (10.2)	0.552
Duration of admission (days)	4 (2–7)	5 (3–8)	3 (2–6)	0.013

ALT, alanine transaminase; AST, aspartate transaminase; BC, blood culture; PCR, real‐time PCR.

a Comparison of patients with confirmed typhoid fever and those without typhoid fever.

b Recent antimicrobial reliably active against locally circulating *S*.Typhi strains (ceftriaxone or azithromycin).

### Rapid diagnostic tests for typhoid fever

A total of 124 of the 300 febrile patients had a positive result with the LifeAssay test with a reading of 1+ in 76 patients and ≥2+ in 48 patients. The CTK RDT was positive in 90/300 patients, positive ≥1+ for IgG in 89 patients and positive for IgM in one patient. The SD Bioline RDT was positive in 20/300 patients, positive for IgM only in 11 patients, positive for IgM and IgG in three patients and positive for IgG only in six patients. The LifeAssay RDT was positive in 30 of the 38 healthy control subjects (29 at a score of 1+ and one at score of 3+). The CTK and SD Bioline RDTs were negative in all the healthy control subjects.

The sensitivity, specificity, and the positive and negative predictive values of the three typhoid fever RDTs are shown in Table 2 using the 34 patients with blood culture and/or PCR‐confirmed diagnosis of typhoid fever as the composite reference standard. The calculated blood culture sensitivity was 55.9%, and the PCR sensitivity was 52.9%. The sensitivity of all RDTs was low. The highest sensitivities were 58.8% with the LifeAssay (IgM) test with the threshold set at ≥1+ and the CTK test (IgM and/or IgG positive), but the specificities were only 60.9% and 73.7%, respectively. When the LifeAssay RDT threshold was set at ≥2+, the sensitivity was 41.2% and specificity was 87.2%. The highest specificities were observed with the SD Bioline RDT (>95%), but the test lacked sensitivity (<25%).

**Table 2:** Conventional sensitivity, specificity, positive and negative predictive values for each laboratory method (blood culture, PCR and each rapid diagnostic test). Laboratory‐confirmed typhoid (blood culture and/or PCR positive; *n* = 34) was the composite reference standard and was compared with cases that were not typhoid based on laboratory confirmation (*n* = 266) (values are means with 95% confidence intervals)

Diagnostic test	Test positive in typhoid cases (*n* = 34)	Test positive in not typhoid cases (*n* = 266)	Sensitivity (95%CI)	Specificity (95%CI)	PPV (95%CI)	NPV (95%CI)
Blood culture	19	0	55.9 (39.4–71.1)	100 (98.3–100)	100 (80.2–100)	94.7 (91.3–96.8)
PCR	18	0	52.9 (36.7–68.6)	100 (98.3–100)	100 (79.3–100)	94.3 (90.9–96.5)
LA (IgM) ≥1+	20	104	58.8 (42.2–73.7)	60.9 (54.9–66.6)	16.1 (10.6–23.7)	92.1 (87.0–95.3)
LA (IgM) ≥2+	14	34	41.2 (26.3–57.8)	87.2 (82.6–90.7)	29.2 (18.2–43.3)	92.1 (88.0–94.9)
CTK (IgM)	0	1	0 (0–12.1)	99.6 (97.7–100)	0 (0–83.3)	88.6 (84.5–91.8)
CTK (IgG)	20	69	58.8 (42.2–73.7)	74.1 (68.5–79.0)	22.5 (15.0–32.3)	93.4 (89.1–96.1)
CTK (IgM &/or IgG)	20	70	58.8 (42.2–73.7)	73.7 (68.1–78.6)	22.2 (14.8–31.9)	92.3 (89.0–96.1)
SD Bioline (IgM)	7	7	20.6 (10.1–37.1)	97.4 (94.6–98.8)	50.0 (26.8–73.2)	90.1 (86.6–93.5)
SD Bioline (IgG)	3	6	8.8 (2.3–23.8)	97.7 (95.1–99.1)	33.3 (11.7–64.9)	89.4 (85.2–92.4)
SD Bioline (IgM &/or IgG)	8	12	23.5 (12.2–40.2)	95.5 (92.2–97.5)	40.0 (21.8–61.4)	90.7 (86.7–93.6)

### Evaluation using a Bayesian latent class model

We applied Bayesian LCMs to explore the sensitivity and specificity of blood culture, real‐time PCR and the various RDTs for typhoid fever diagnosis. We ran the model with the LifeAssay RDT threshold set at ≥1+ and then again with the threshold set at ≥2+ (Table 3). The specificity of blood culture was set at 100%. The sensitivities and specificities of all tests apart from the LifeAssay RDT were consistent between the two runs as expected. The sensitivity of blood culture was 55.6% and 47.5% in the two model runs, and the sensitivity of the real‐time PCR was only 14.2%. The best combination of sensitivity and specificity was observed with the LifeAssay RDT with the threshold set at ≥2+ at 63.0% and 91.2%, respectively. The SD Bioline specificity was higher than the other RDTs (>95%) but with a sensitivity of <40%. The calculated prevalence in the two model runs was 11.8% and 13.6% consistent with the observed prevalence of 11.3% based on laboratory‐confirmed cases.

**Table 3:** Bayesian LCM model prevalence sensitivity and specificity for the results of blood culture, real‐time PCR and each individual rapid diagnostic test. The specificity of blood culture was set at 100%. Results are medians with 95% credible intervals. The model was run with the Life Assay (IgM) threshold set at ≥1+ and again with the Life Assay (IgM) threshold set at ≥2+

Diagnostic test	Parameter	Model run using Life Assay (IgM) ≥1+ as positive cut‐off	Model run using Life Assay (IgM) ≥2+ as positive cut‐off
	Prevalence	11.8 (6.7–19.5)	13.6 (8.1–22.1)
Blood Culture	Sensitivity	55.6 (27.6–87.7)	47.5 (23.2–76.9)
	Specificity	100	100
PCR	Sensitivity	14.2 (2.8–32.8)	14.2 (3.0–32.0)
	Specificity	95.0 (91.8–97.3)	95.3 (92.1–97.6)
Life Assay (IgM)	Sensitivity	86.7 (68.6–96.8)	63.0 (43.3–80.8)
	Specificity	64.8 (58.4–71.3)	91.2 (86.5–96.0)
CTK (IgM &/or IgG)	Sensitivity	60.5 (40.3–79.1)	60.8 (41.8–78.9)
	Specificity	73.9 (68.2–79.4)	74.7 (68.8–80.3)
SD Bioline ((IgM &/or IgG)	Sensitivity	37.4 (20.0–57.2)	36.0 (20.3–55.8)
	Specificity	97.1 (94.0–99.4)	97.6 (94.7–99.6)

If the proportion of positive samples for each test at different durations of illnesses prior to admission is considered, PCR was more frequently positive in the initial three days of illness, whereas blood cultures were only positive in patients with four or more days of fever.

## Discussion

The primary finding of this study was that the diagnostic accuracy of three RDTs for typhoid fever in febrile hospitalised patients in Chittagong, Bangladesh, was only moderate. The sensitivity results of the three tests ranged from 24% to 59% with specificities of 61–96%. In the Bayesian LCM, the sensitivity of the Life Assay RDT was higher at 87% but with a specificity of only 65% and the sensitivity and specificity of the other two RDTs broadly similar. The model estimates for prevalence of typhoid were close to the observed prevalence of 11.3%, based on laboratory‐confirmed cases. The three typhoid RDTs were also tested in healthy adult controls subjects. The CTK and SD Bioline RDTs gave negative results, but with the LifeAssay RDT 30 of the 38 controls, subjects gave a positive result (29 at 1+). In the light of this, the LifeAssay RDT results were re‐analysed with a threshold of ≥2+ rather than ≥1+ to indicate a positive result and the sensitivity fell from 59% to 41% but the specificity rose from 61% to 87%. When the Bayesian LCM was rerun using this threshold, the sensitivity of 63% and specificity of 91% was the best result combination of all the three RDTs.

The LifeAssay RDT format has been studied in 209 febrile patients presenting to hospital in Makassar, Indonesia, in whom 116 (55.5%) were diagnosed with typhoid fever, 54 (25.8%) confirmed by blood culture 24. The sensitivity and specificity (with an intensity of ≥1+ as positive) was 62.1% and 97.8%, respectively. A further study, conducted in Cambodia, of 500 hospitalised febrile children included 44 (8.8%) cases of typhoid fever, of which 32 (6.4%) were confirmed by blood culture and/or real‐time PCR 15. The sensitivity and specificity (with a threshold of ≥2+ considered positive) was 55% and 98%, respectively. When assessed using LCM analysis, the sensitivity of the LifeAssay RDT increased to 77% and the specificity was 98%. A cut‐off threshold of ≥2+ could be reliably determined with high inter‐ and intra‐observer kappa scores 15.

The SD Bioline RDT was studied in 177 febrile hospitalised children and adults in the Philippines of which 75 (39.5%) were blood culture positive for *S. *Typhi 25. The sensitivity and specificity was 69% and 79%, respectively, for IgM and 71% and 76%, respectively, for IgG. These sensitivities are higher than the results presented here. We are unaware of any published evaluations of the CTK RDT in febrile patients.

The sensitivity of blood culture in this study was relatively low at 56% of laboratory‐confirmed cases and 48–56% in the LCMs. This is comparable to studies where bone marrow culture was additionally performed 26, 27, 28, 29. Considerable efforts were made to take large and reproducible blood volumes for culture in this study. The recent consumption of antimicrobials was commonly reported in all groups although we cannot be sure that the drugs taken were as described or of the required quality to be effective. In the study in Cambodian children using LCMs to evaluate diagnostic tests for typhoid, blood culture sensitivity approached 80%, despite lower volumes of blood, although a lower number of patients reported recent antimicrobial use 15.

The proportion of positive cases positive by both blood culture and PCR was low. The lack of PCR‐positive samples from patients with a positive blood culture may relate to the smaller volume of blood used to extract the DNA for the PCR compared with that used for the blood culture and also the potential presence of PCR inhibitors. The PCR‐positive/blood culture‐negative patients may be the result of prior antimicrobial therapy. The proportion of blood culture negative/PCR‐positive patients who had taken an antimicrobial active against local strains of *S*. Typhi was higher than those with a positive blood culture, but the numbers were small and not significant. The PCR was often positive in the first few days of fever, a similar observation to the study in Cambodia 15. There may be an early bacteraemiac period in which the organism is difficult to culture and this is an area that warrants further study.

The inclusion of consecutive patients in this endemic setting makes the findings more generalisable for hospitalised patients with fever although may not apply to patients in ambulatory settings. All diagnostic tests were performed on samples collected at the time, or within 48 h, of admission, and the reference and index tests were performed blind. The small number of laboratory‐confirmed typhoid cases is a limitation resulting in large confidence intervals for the diagnostic accuracy estimates. All patients received the same diagnostic composite reference standard although this reference standard is not perfect. Some of the patients with clinically suspected typhoid fever who were blood culture and PCR negative may have been true positives. Analysis with Bayesian LCMs attempts to address this issue. There were few patients with bacteremia caused by alternative organisms and therefore limited opportunity to assess potential cross‐reactivity of these RDTs with other causes of bacteremia.

## Conclusions

There is no consensus as to the required target product profile for a RDT to diagnose acute typhoid fever. In our opinion, cut‐offs of >80% for sensitivity and >98% for specificity would be reasonable targets. None of the RDTs tested reach these levels of sensitivity and specificity. Similar observations apply to other widely used commercially available typhoid RDTs 13. This does not necessarily mean that tests with these reported sensitivity and specificity values are not useful as long as the positive and negative predictive values of the test are clearly understood by the user. Although larger evaluations of typhoid RDTs in other settings are needed to substantiate these findings, it is clear that new RDTs using more specific antigens against *S. *Typhi and *S. *Paratyphi A are needed 30. The lack of a satisfactory reference standard remains an important limitation of such studies and the place of real‐time PCR in the laboratory confirmation of typhoid fever needs to be further improved and evaluated.

## Appendix 1

### CMCH Typhoid Study Group

Abdullah Abu Sayeed, Mahtab Uddin Hasan, Wirichada Pan‐Ngum, Thomas W. van der Vaart, Asok Kumar Dutta, Nasir Uddin Mahmud, Murad Hero, Nafiz Iqbal, Zabeen Chaudhury, Tran Vu Thieu Nga, Pham Thanh Duy, Voong Vinh Phat, Richard J. Maude, Stephen Baker, W. Joost Wiersinga, Tom van der Poll, Nicholas P. Day, Arjen M. Dondorp.
